# Novel Blood Biomarkers for Response Prediction and Monitoring of Stereotactic Ablative Radiotherapy and Immunotherapy in Metastatic Oligoprogressive Lung Cancer

**DOI:** 10.3390/ijms25084533

**Published:** 2024-04-20

**Authors:** Juan Zafra, Juan Luis Onieva, Javier Oliver, María Garrido-Barros, Andrea González-Hernández, Beatriz Martínez-Gálvez, Alicia Román, Rafael Ordóñez-Marmolejo, Elisabeth Pérez-Ruiz, José Carlos Benítez, Andrés Mesas, Andrés Vera, Rodolfo Chicas-Sett, Antonio Rueda-Domínguez, Isabel Barragán

**Affiliations:** 1 Group of Translational Research in Cancer Immunotherapy (CIMO2), Department of Radiation Oncology, Virgen de la Victoria University Hospital, Institute of Biomedical Research in Malaga (IBIMA), 29010 Málaga, Spain; jzafra08@gmail.com; 2 Faculty of Medicine, University of Malaga (UMA), 29071 Málaga, Spain; juanluonieva@gmail.com (J.L.O.); mariagarridobarros@gmail.com (M.G.-B.); guenia99@gmail.com (A.G.-H.); 3 Group of Translational Research in Cancer Immunotherapy (CIMO2), Medical Oncology Intercenter Unit, Regional and Virgen de la Victoria Hospitals, Institute of Biomedical Research in Malaga (IBIMA), 29010 Málaga, Spain; javiom@gmail.com (J.O.); beamartg@gmail.com (B.M.-G.); eliperu@gmail.com (E.P.-R.); jcbenitezhuvv@gmail.com (J.C.B.); 4 Department of Radiation Oncology, Virgen de la Victoria University Hospital, Institute of Biomedical Research in Malaga (IBIMA), 29010 Málaga, Spain; aliciajobac@hotmail.com (A.R.); rafaelordm@gmail.com (R.O.-M.); 5 Medical Oncology Intercenter Unit, Regional and Virgen de la Victoria Hospitals, 29010 Málaga, Spain; mesasruiz91@gmail.com; 6 Department of Radiation Oncology, Dr Negrín University Hospital, 35010 Las Palmas de Gran Canaria, Spain; andresvera1592@gmail.com; 7 Department of Radiation Oncology, La Fe University Hospital, 46026 Valencia, Spain; rchicas@ufm.edu; 8 Group of Clinical and Translational Cancer Research, Le Fe Health Research Institute, 46026 Valencia, Spain; 9 Group of Pharmacoepigenetics, Department of Physiology and Pharmacology, Karolinska Institutet, 171 77 Stockholm, Sweden

**Keywords:** immunotherapy, stereotactic ablative radiotherapy, oligoprogression, biomarkers, cell-free DNA, small RNA

## Abstract

Up to 80% of patients under immune checkpoint inhibitors (ICI) face resistance. In this context, stereotactic ablative radiotherapy (SABR) can induce an immune or abscopal response. However, its molecular determinants remain unknown. We present early results of a translational study assessing biomarkers of response to combined ICI and SABR (I-SABR) in liquid biopsy from oligoprogressive patients in a prospective observational multicenter study. Cohort A includes metastatic patients in oligoprogression to ICI maintaining the same ICI due to clinical benefit and who receive concomitant SABR. B is a comparative group of oligometastatic patients receiving only SABR. Blood samples are extracted at baseline (T1), after the first (T2) and last (T3) fraction, two months post-SABR (T4) and at further progression (TP). Response is evaluated by iRECIST and defined by the objective response rate (ORR)—complete and partial responses. We assess peripheral blood mononuclear cells (PBMCs), circulating cell-free DNA (cfDNA) and small RNA from extracellular vesicles. Twenty-seven patients could be analyzed (cohort A: n = 19; B: n = 8). Most were males with non-small cell lung cancer and one progressing lesion. With a median follow-up of 6 months, the last ORR was 63% (26% complete and 37% partial response). A decrease in cfDNA from T2 to T3 correlated with a good response. At T2, CD8+PD1+ and CD8+PDL1+ cells were increased in non-responders and responders, respectively. At T2, 27 microRNAs were differentially expressed. These are potential biomarkers of response to I-SABR in oligoprogressive disease.

## 1. Introduction

Immune checkpoint inhibitors (ICI) have transformed the treatment landscape for patients with immunogenic tumors such as non-small-cell lung cancer (NSCLC), achieving significant improvements in survival and with a favorable toxicity profile [1]. However, only 20–30% initially benefit, and a relevant number of responders end up developing secondary resistances [2]. It has been suggested that this resistance is partially explained by these being “cold” tumors, which lack T-cell infiltration [3]. To overcome these challenges, new strategies are arising, such as the combination of radiotherapy (RT) with ICI.

RT can induce an immune response able to unleash antitumor effects outside the radiation field. This phenomenon, known as the abscopal response (AR), had traditionally been considered extraordinarily infrequent [4]. However, recent randomized studies have evidenced better response rates with the addition of RT to ICI, especially in the form of stereotactic ablative radiotherapy (SABR) [5]. Moreover, there is solid evidence that SABR is effective in oligometastatic disease (OMD). OMD has been defined as an intermediate state between localized and metastatic disease in which locally ablative therapies (alone or in combination with systemic therapy) may be potentially curative [6]. Current international guidelines such as the consensus by the American Society of Radiation Oncology (ASTRO) and the European Society of Radiation Oncology (ESTRO) identify oligometastatic patients as those with up to five lesions, independently of the primary tumor [6]. A particular form of OMD is oligoprogression, which refers to a limited progression of one or few lesions during active systemic therapy. This oligoprogression can be present in an already oligometastatic patient or be “induced” by systemic therapy in a polymetastatic patient. In this context, SABR is useful for maintaining the same line of systemic therapy and avoiding a change to less effective treatments [7]. In NSCLC with driver mutations, strong responses that allow for the continuation of targeted therapy have been reported after the addition of SABR [8].

Considering the above, the role of SABR in the setting of oligoprogression to ICI is especially interesting based on the synergies between SABR and ICI (I-SABR). It has been reported that the effects of RT can turn “cold” tumors into “hot” ones by improving T-cell activation and infiltration [9]. To this end, a recent prospective study conducted by our group in oligoprogressive patients with NSCLC and melanoma added SABR to progressing sites [10] and reported an objective response rate (ORR) of 42% and 65% AR, with a promising median progression-free survival (PFS) of 14.2 months that allowed for the continuation of the same ICI. However, not all patients responded to this approach, and no distinct clinical characteristics that explained this difference in response were found. Moreover, several studies have reported no benefit with the addition of RT to ICI [11,12]. For these reasons, there is a need for predictive and prognostic biomarkers that can guide patient selection and establish the mechanisms of AR in humans [13].

Given the difficulty of accessing solid tumor tissue, especially in metastatic patients, liquid biopsy has become increasingly important for biomarker analysis [14]. Circulating tumor DNA (ctDNA) has been proposed as a prime candidate for a predictive biomarker of response [15] but can be difficult and expensive to identify [16]. By contrast, cell-free DNA (cfDNA) is released by normal blood cells, primary lesions and metastases, and circulating tumor cells. It is associated with tumor burden and advanced stage [17]. In a previous report, it has been described how the cfDNA concentration can predict the response to ICI in NSCLC [18], but its association with SABR has not been studied. Peripheral blood mononuclear cells (PBMCs) may also be useful to trace AR, as CD8+ T cells have been associated with RT response [19]. Furthermore, small RNA contained in extracellular vesicles (EVs) circulating in the blood are responsible for intercellular communication [20] and are rapidly influenced by both RT [21] and ICI [22]. Differences in expression may be able to predict the treatment response.

We present preliminary results of a prospective and translational study of I-SABR in patients with oligoprogressive metastatic cancer maintaining the same line of ICI due to clinical benefit. Blood samples for cfDNA and dynamic EV analyses were gathered at several time points before, during and after treatment to correlate them with clinical variables of response.

## 2. Results

### 2.1. Baseline Characteristics

From 1 November 2021 to 30 November 2023, 55 patients were recruited for the study. For this first analysis, we included the 27 patients who had at least one imaging reevaluation after SABR by the cut-off date of 10 July 2023 (19 from cohort A and 8 from B). None of these patients were lost during follow-up. Patient and treatment characteristics are detailed in Table 1 (cohort A) and Table 2 (cohort B). The median age, sex distribution and ECOG were similar between cohorts, with a predominance of males (73.7% in A and 65% in B). All patients had an ECOG of 0 or 1. Lung cancer was the most frequent in both cohorts (73.6% in A and 50% in B), followed by renal cancer (15.8% and 25%, respectively). In cohort A, 52.6% of patients were polymetastatic compared to only 12.5% in cohort B. The PD-L1 status was unknown in most patients. Only one patient (cohort A) had a driver mutation (BRAF). In cohort A, 42.1% had received prior RT compared to 50% in B. As for previous systemic therapy for metastatic disease, 36.8% in cohort A had received up to one line. In cohort B, 25% had received one line and 12.5% three lines. The most frequent treatment site was the lungs for both groups (40% and 60%), and most patients were treated for one lesion (62% and 75%). Ninety-eight percent of lesions in cohort A were treated with 35 Gy/5 fx. In cohort B, the most frequent ablative dose was 50 Gy/5 fx. Three patients in cohort A received SABR to a partial volume due to large size that limited dose constraints. Up to this point, no patient had been rechallenged with further SABR courses.

### 2.2. Clinical Outcomes

With a median follow-up of 6 months (range, 3.4–19.8 months), the last ORR considering both cohorts was 63%: 26% CR (n = 7) and 37% PR (n = 10). Furthermore, 3.7% (n = 1) had stable disease (SD) and 33.3% (n = 9) suffered new progression. Treatment results in each cohort are displayed Table 3. AR could be measured in 14 patients in cohort A (the 5 missing patients had all their measurable lesions irradiated and, therefore, could not be evaluated for this endpoint), which was 35.7% (n = 5). LC was 88% (n = 22 lesions) in cohort A and 100% (n = 10 lesions) in cohort B. The three metastases that progressed locally after SABR were all bulky masses that had to be treated with partial irradiation to comply with dose constraints. These three patients also developed rapid disease progression globally. Of the 9 patients who further progressed, 8 commenced a new line of systemic therapy (5 of them with immunotherapy combined with new agents as part of clinical trials).

**Figure 1:**
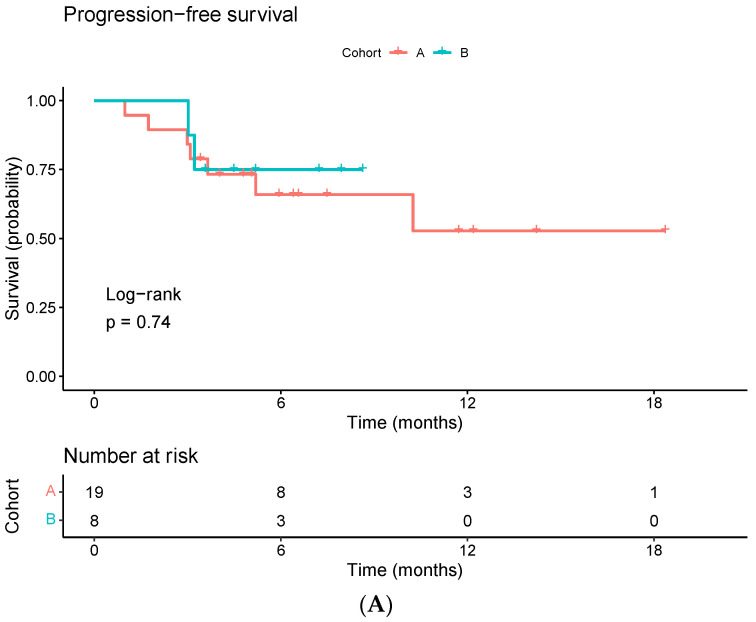

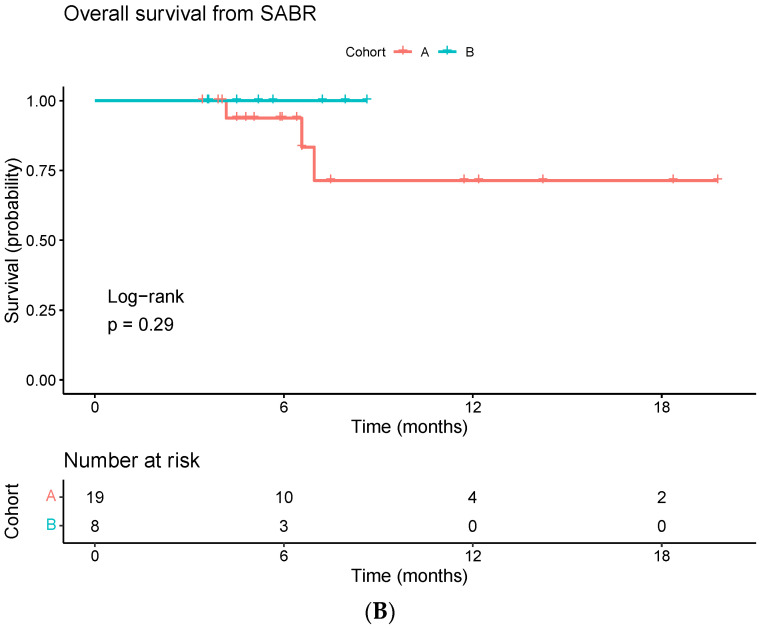
Kaplan–Meier curves for progression-free (**A**) and overall survival (**B**).

Preliminary survival analysis is displayed in Figure 1. Kaplan–Meier curves showed no significant differences in PFS or OS between groups. Median OS and PFS have not been reached. At the time of analysis, 88% of patients (n = 24) were alive. Of the three patients who died (all from cohort A), two had non-squamous NSCLC and one squamous cervix cancer. Save for one patient with NSCLC who initially responded to I-SABR but suffered from multiple brain progression 6 months after, the other two patients had bulky masses of more than 10 cm treated with partial SABR and experienced both local and distant progression, dying shortly after. No patients died before the first imaging evaluation after SABR.

In terms of acute toxicity, 47.4% (n = 9) of patients in cohort A experienced grade 1–2 toxicity after I-SABR, the most frequent being local pain at the irradiated site (n = 5). In cohort B, 37.5% (n =5) experienced grade 1–2 toxicity. All these toxicities had been resolved by the cut-off date for this analysis. No grade ≥ 3 side effects were reported. No immune-related adverse events were observed in cohort A, and SABR did not cause systemic therapy interruptions in any patient.

### 2.3. Liquid Biopsy Assessment

**Figure 2:**
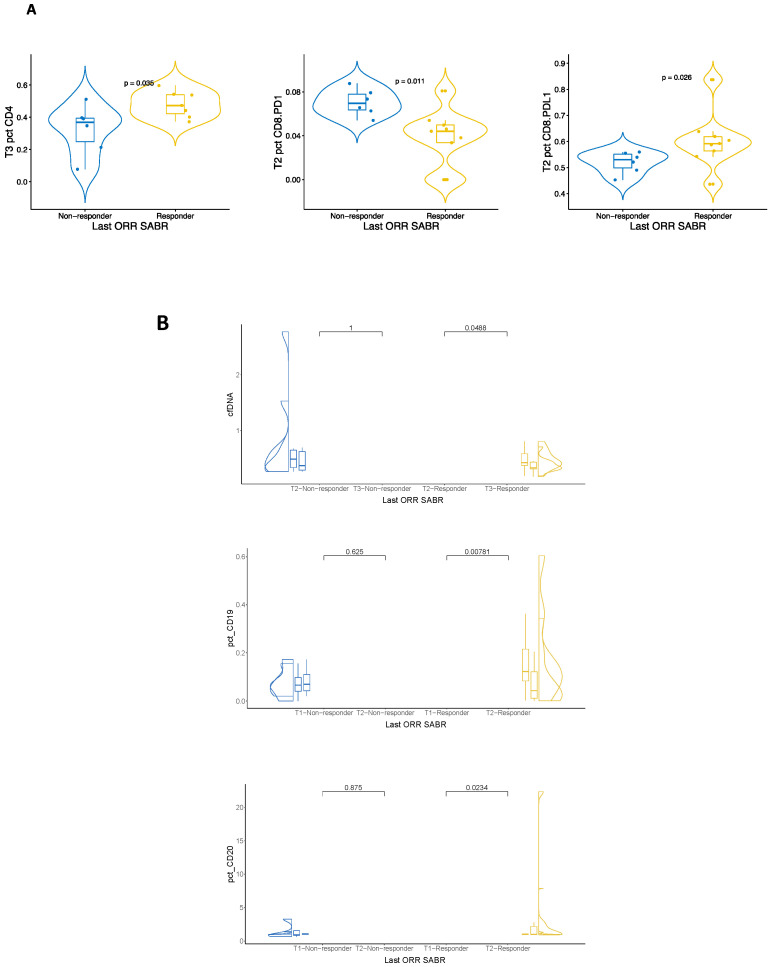
(**A**) Univariate analysis of significant differences between responders and non-responders in terms of cell-free DNA concentration and percentage of PMBCs. (**B**) Longitudinal analysis of significant differences in responders and non-responders. Blue dots represent samples from non-responders and orange dots from responders.

Patients were classified as responders or non-responders in terms of the last ORR, and this was correlated with the molecular findings across all time points. In this paper, we describe those time points and longitudinal changes that were statistically significant. Fifteen patients with NSCLC were available for cfDNA and PMBCs studies. In univariate analysis (Figure 2A), the percentage of CD8+PD1+ cells was significantly higher in non-responders vs. responders at T2 (*p* = 0.011). In contrast, CD8+PDL1+ cells were higher in responders (*p* = 0.026). Moreover, at T3, the percentage of CD4+ was significantly higher in responders compared to non-responders (*p* = 0.036). In paired longitudinal analysis (Figure 2B), the concentration of cfDNA significantly decreased in responders from T2 to T3 (*p* = 0.0488) but did not vary in non-responders. From T1 to T2, the percentage of CD19+ cells significantly decreased in responders (*p* = 0.00781), while CD20+ significantly increased (*p* = 0.0234). These changes were not observed in non-responders.

**Figure 3:**
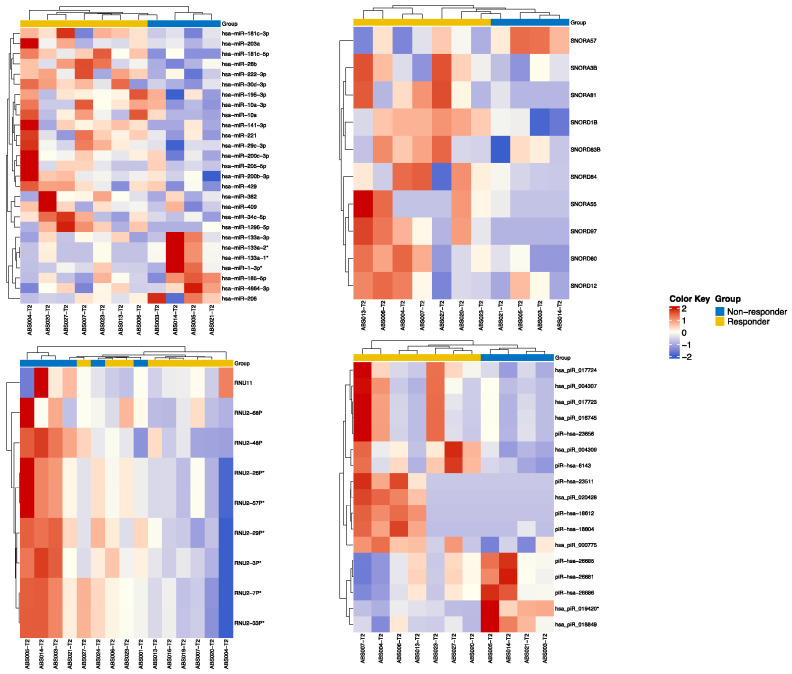
Heatmap for differential expression at T2 between responders and non-responders in terms of significant microRNA (miRNA), small nucleolar RNA (snoRNA), small nuclear RNA (snRNA) and piwi-interacting RNA (piRNA). All molecules displayed exhibited differential expression with a *p* value < 0.05. Small RNAs with an asterisk (*) represent those that are also significant with adjusted *p* value < 0.05. Blue tones represent downregulation and red tones represent upregulation.

**Figure 4:**
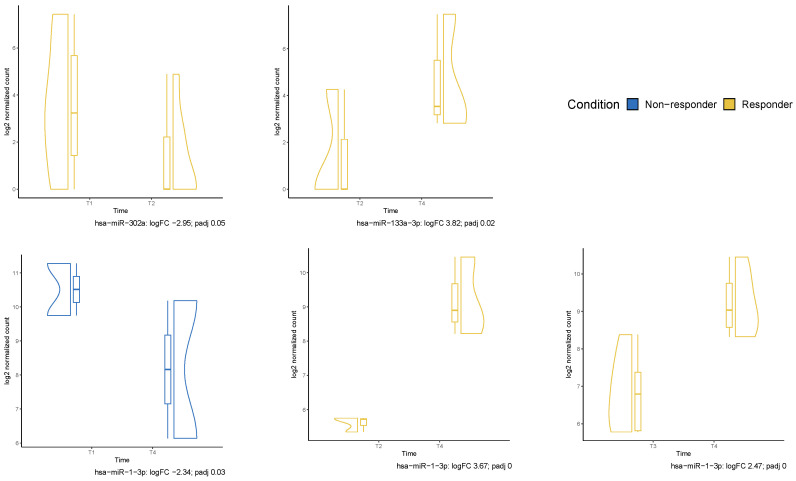
Longitudinal analysis of significantly differentially expressed miRNAs between time points.

**Figure 5:**
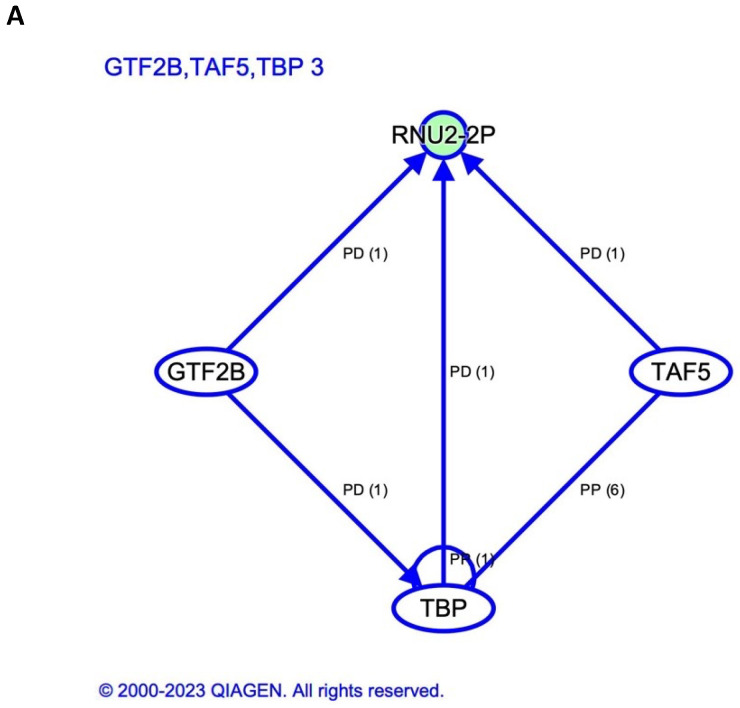

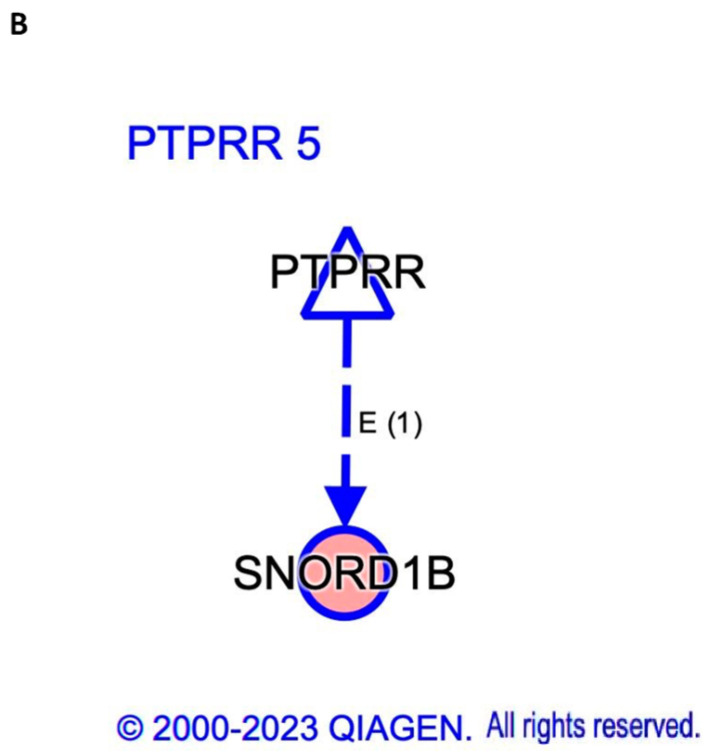
Graphical representation of the upstream analysis of relevant small RNA in T2 according to ingenuity pathway analysis. (**A**) RNU-2P expression is influenced by TBP, TAF5 and GTF2B. (**B**) SNORD1B pathway is influenced by PTPRR. Abbreviations: PD = protein–DNA binding; E = expression; PP: protein–protein binding; TAF5 = TATA-box binding protein associated factor 5; TBP = TATA-box binding protein 3, GTF2N = general transcription factor IIB.

Small RNA data for differential expression analysis between responders and non-responders (last ORR) were available from T1 to T4 samples of 14 patients with NSCLC in cohort A. Two patients were excluded due to being outliers. At T2, we found 27 miRNAs that were differentially expressed between responders and non-responders with *p* value < 0.05. Of these, three (hsa-miR-133a-1, hsa-miR-133a-2 and hsa-miR-1-3p) were significantly downregulated in responders with adjusted *p* value < 0.05. Figure 3 shows this heat map, together with those for small nucleolar RNA (snoRNA), small nuclear RNA (snRNA) and piwi-interacting RNA (piRNA). RNU2-57P, RNU2-29P, RNU2-3P, RNU2-7P, RNU2-33P and hsa-piR-019420 were all significantly downregulated in responders with an adjusted *p* value. As for longitudinal analysis, hsa-miR-302a became significantly downregulated from T1 to T2 in responders. hsa-miR-1-3p became upregulated in responders from T2 to T4 and from T3 to T4 but downregulated in non-responders from T1 to T4. Finally, hsa-miR-133a-3p became upregulated from T2 to T4 in responders (Figure 4). IPA software (version 01-23-01) identified four upstream regulators related to small RNA in T2. TATA-box binding protein associated factor 5 (TAF5), TATA-box binding protein (TBP) and general transcription factor IIB (GTF2B) were associated with RNU2-2P, which was downregulated in responders. In contrast, PTPRR was associated with SNORD1B and upregulated in responders (Figure 5). No differences in small RNA or cfDNA-PBMC studies were identified between patients with squamous and non-squamous NSCLC.

## 3. Discussion

Although I-SABR is a promising approach in patients with oligoprogressive disease, the determinants of response remain unknown. Biomarkers in liquid biopsy may be helpful to understand the AR and generate predictive and prognostic models. In terms of clinical response, our results seem comparable to previously published data. Last ORR in cohort A was slightly higher compared to our previous study on a very similar patient population [10] (57.9% vs. 42%), but this is probably due to a much shorter follow-up in this analysis. A recent pooled analysis of two phase II trials has reported a 62.5% disease control rate at 3 months and median PFS of 4.4 months in patients with oligometastatic (1–4 lesions) NSCLC in progression to ICI [7]. In contrast, the randomized phase II STOP trial did not achieve its PFS endpoint. Patients with oligoprogressive disease to 1–5 lesions were randomized to receive SABR to all sites plus systemic therapy vs. systemic therapy alone. With a median follow-up of 31 months, there was no significant difference in PFS between arms. As pointed out by the authors, these results might be explained by suboptimal protocol adherence in the standard of care arm and by the fact that crossover was permitted between arms, which could be a determining confounding factor. Moreover, only 27% had ICI as systemic therapy, which greatly differs from our current study and might reflect a larger benefit from the combination with immunotherapy [12]. Recently, the phase II randomized CURB trial published its first results in patients with oligoprogressive breast cancer and NSCLC. Interestingly, there was no difference in PFS between arms in the breast cancer cohort, but the median PFS in the NSCLC groups was more than four times higher in the SBRT arm compared to the standard of care arm (10 months vs. 2.2 months; HR 0.41; *p* = 0·0039) [23]. These observations further point out the importance of patient selection to optimize results. While cohort B is probably not clinically comparable to A given the differences in the natural history of the disease (patients in A have poorer prognosis), interesting comparisons can be analyzed in terms of biomarker expression once we have a larger sample size.

In our study, AR in evaluable patients was 35.7%, which is considerably lower compared to our previous study (65%) [10], but still higher than most published data from other authors [5,12]. In the first study, 38% of patients had metastatic melanoma vs. none in this analysis. This may be a more immunogenic tumor type and, therefore, more prone to present AR. PFS and OS are not comparable yet due to the short follow-up, but the fact that medians have not been reached suggests that I-SABR is useful to extend the clinical benefit of ICI as previously reported [7,10]. Given the good results in terms of response, LC and safety with subablative doses [10], we decided to use the same SABR fractionations for the present study. We are aware that other groups have employed higher doses with comparable results [24]. Nonetheless, the most immunogenic fractionation is yet to be discovered.

The concentration of cfDNA as a biomarker has never been analyzed in the context of I-SABR. A recent study communicated in ASTRO assessed ctDNA in oligometastatic patients with NSCLC receiving SABR and ICI [15]. In this study, it was found that baseline ctDNA correlated with both PFS and OS (hazard ratio 3.781 and 5.423, respectively), whereas the number of metastatic organ systems did not. Moreover, the CURB trial found that patients with NSCLC who received SABR plus systemic therapy had a significant decrease in ctDNA between baseline and follow-up, whereas the standard of care only group showed no significant differences [23]. If cfDNA was found to be a surrogate for ctDNA, it would make it a much simpler and widely available technique. We observed a decrease in cfDNA across the SABR treatment (from T2 to T3) in responders. The concentration at baseline (T1) was not significantly different between groups (*p* = 0.62). In a previous work regarding patients with newly diagnosed metastatic NSCLC with only systemic therapy (only ICI or combinations), low cfDNA at baseline was predictive of good response [18]. For our study, a larger number of patients and further follow-up are needed to determine if this difference also exists in the oligoprogressive setting.

In terms of PBMCs, we found a significantly lower percentage of CD4+ cells in non-responders compared to responders. This is in line with several studies suggesting poor response and survival in patients with NSCLC and CD4+ lymphopenia [25]. Interestingly, SABR in less than 10 Gy per fx for early-stage NSCLC has been reported to increase the fraction of proliferating CD4+ and CD8+ circulating T-cells, which may contribute to a systemic immune response [26]. At T2, CD8+PD1+ cells were significantly decreased and CD8+PDL1+ increased in responders. Both findings are consistent with previously reported data in NSCLC. A reduction in PD1+ circulating CD8+ T cells after the start of ICI has been associated with better response and survival [27]. In contrast, reduced circulating CD8+PDL1+ cells are related to worse outcomes [28]. In longitudinal analysis, responders showed decreased CD19+ and increased CD20+ B cells after the start of SABR. CD20+ cells positively correlate with response and survival [29]. cfDNA and PBMCs offer the advantage of being inexpensive and easy to determine in a standard laboratory, making them promising biomarkers for their use in daily clinical practice. Interestingly, our analyses suggest that these are early biomarkers, which means that they could potentially anticipate the response observed in post-treatment imaging.

Pathway analysis in T2 samples of NSCLC patients from cohort A (Figure 3) yielded two small RNA with upstream regulators (Figure 5). RNU2-2P is a pseudogene that is involved in cancer [30] and is inhibited by TBP, TAF5 and GTF2B. Although these have not been previously described in NSCLC, high expression of GTF2B has been associated with a good response in breast cancer [31]. Furthermore, high expression of TAF5 has been associated with a good response in gastric cancer [31]. The second small RNA, SNORD1B, has been described to be downregulated in NSCLC compared to healthy donors [32]. Interestingly, in our study, this small RNA was upregulated in responders, which could be indicative of an effective tumor response induced by SABR. RNU2-57P, RNU2-29P, RNU2-3P, RNU2-7P, and RNU2-33P are all part of this snRNA family and were also downregulated in responders. Moreover, high expression of PTPRR, which is involved in this pathway, has been described in several types of cancer [33]. Other significant small RNAs at T2 in our study are backed by the literature. hsa-miR-1-3p has been described as a key contributor to the progression of NSCLC via the regulation of mitosis [34]. This miRNA was significantly upregulated in non-responders, which is consistent with these previous findings. Of note, hsa-miR-133a-1 and hsa-miR-133a-2 were downregulated in responders and upregulated in non-responders. This contradicts previously published data on the hsa-miR-133a family being downregulated in several cancers, including NSCLC, and a potential biomarker of favorable response [35]. We hypothesize that this may be a temporary finding associated with the first SABR fraction, as this difference in expression was not observed at other timepoints. However, as the role of RT in the expression of this miRNA has not been established in any other studies, we cannot reach any conclusions in this regard. Another significant small RNA in our cohort, hsa-piR-019420, has been reported to be upregulated in patients with hepatocellular carcinoma compared to healthy controls [36]. While this has not been described as being different among cancer patients with different types of response, we found it to be downregulated in responders. This downregulation, as it has been seen in healthy patients, may be indicative of a favorable tumor response in our cohort.

Longitudinal analyses offer the opportunity to assess the evolution of biomarkers over time and in response to treatment (Figure 4). In this regard, hsa-miR-1-3p became significantly upregulated in responders from T2 to T3 and from T2 to T4 and downregulated in non-responders from T1 to T4. Despite these oscillations, this miRNA was persistently higher in non-responders throughout treatment, consistent with its known association with tumor progression [34]. Moreover, those oscillations bring about possible hypotheses regarding the reprogramming induced by radiation.

Assessing these readouts longitudinally was necessary to reveal several miRNAs informative of disturbances that affect only one group of patients. These variations can be specifically related either with resistance or response mechanisms. For example, hsa-miR-302a expression is not different when comparing responders versus non-responders at any timepoint but experiences a temporary decrease when comparing pre-treatment (T1) to post-first SABR fraction (T2) time points. While the relevance in long-term response is not established, this finding is important for understanding tumor processes that may occur early after irradiation in the context of a tumor previously exposed to ICI. Interestingly, the downregulation of hsa-miR-302a has been associated with radiotherapy resistance and a poor response in lung cancer [37], so this temporary change could represent an initial refractory reaction to radiation.

Another small RNA that fluctuated according to the response during treatment is hsa-miR-133a-3p. While after the first fraction (T2) the static comparison of its expression indicated overexpression in non-responders, from that point to two months after (T4), responders had increasing concentrations of this transcript. In this case, the continuous increment is coherent with the reported role of hsa-miR-133a-3p against lung adenocarcinoma development and chemotherapy resistance [38].

We are not aware of any reports on significant EVs with the combination of SABR and ICI. Moreover, small RNA analysis as related to RT is a very novel field, though some authors have published data on potential biomarkers [39]. A recent study on patients receiving concurrent RT and chemotherapy for locally advanced NSCLC identified three potential biomarkers: miR-375, miR-200c and miR-30c. These are cancer suppressors that were downregulated at baseline in non-responders [40]. While not statistically significant, we did find similar results in our cohort: miR-375 was downregulated in non-responders and upregulated in responders. Interestingly, miR-375 was statistically significant in one of our previous reports with fewer patients [41], so a larger sample size might be more conclusive in the future. Although this is a very novel field and no EVs currently have clinical applications, the fact that we found significant differences in expression as early as T2 reflects their potential as early biomarkers of response that could precede current standard imaging tests.

The present study has several limitations to note. Because of its observational nature, selection bias and confounding factors are issues that can limit validity. We aimed to account for possible biases and confounding factors during the study design and the statistical interpretation, but our recruitment protocol is an inherent source of selection bias. Determining the correct sample size for the desired power in our study was challenging due to the scarce previously published evidence in this setting at the time of design. We recognize that the validation of biomarkers requires large sample sizes, but oligoprogression to ICI is still relatively infrequent in clinical practice. To reach a relevant sample size, we decided to include all cancer types under ICI, even if that meant having more heterogeneous cohorts. However, this was necessary for enough patient recruitment. It must be noted that the design of our study reflects real-world practice, where patients with different types of tumors receive ICI and SABR as part of their standard treatment. Consequently, we believe that the mechanisms of the SABR-induced AR may be shared across tumor types, and basket studies on OMD such as the SABR-COMET trial have found very positive results in different cancer types [42]. For this reason, and even though this first analysis focuses on patients with NSCLC, we aim to find biomarkers of response to I-SABR that may be defining of I-SABR in general and not of a specific tumor type. In addition, we decided to focus on those translational readouts that were significantly different with an adjusted *p* value, in order to increase the stringency of the results. Furthermore, to truly validate future biomarker data, a clinical trial with a larger sample size is under planning to validate the key identified biomarkers once the present study is concluded. An important limitation of this publication is the fact that we did not conduct multivariate analysis given the low sample size and short follow-up. While this could lead to confounding, we thought that reporting the primary endpoint of ORR and its correlation with the molecular analyses was enough for this preliminary report. We expect to assess multivariate analysis in a future report with more patients and longer median follow-up. In this regard, survival data must be taken with caution, as the median follow-up is quite short, and few events have been reported up to the writing of this manuscript. Moreover, the long-term clinical significance of the translational results must be confirmed with a longer follow up. Nonetheless, the time points in which these markers have been analyzed seem to suggest that these could be early indicators of response. Even if this is an interim analysis with findings that must be confirmed at the study close, given the fact that I-SABR has become a reality in clinical practice, there is an urgent need to establish biomarkers that can guide patient selection. We believe that our data, albeit early, is a valuable contribution to the field that can help future research.

A recent report has suggested that high aneuploidy, which is associated with aggressive biology and poor baseline immune infiltration, actually benefits the most from the addition of SABR to standard ICI treatment [43]. We could not directly assess this finding in our patients as solid tissue samples were not available. We were also unable to conduct ctDNA analysis in order to correlate it with our cfDNA results. However, we plan to develop a panel in the future to make this comparison in upcoming samples. With our current data, we were unable to determine any potential predictive biomarkers at baseline, which would be extremely useful for patient selection in this clinical context. Nonetheless, given the many lingering questions on the abscopal response in humans, we believe that our molecular results can contribute to a better understanding of the response to I-SABR. Our results and those from other groups should lead to the development of clinical trials in order to validate these biomarkers. However, smaller studies like ours (both in I-SABR) are important to identify candidate biomarkers in discovery cohorts, given the high number of analytes than can be assessed in liquid biopsy and the high cost of many of these procedures. Once we identify the most promising, these may be validated in larger trials on oligoprogression.

## 4. Materials and Methods

### 4.1. Participants

A prospective observational study in two cohorts was carried out in five hospitals from 1 November 2021. Cohort A consisted of patients ≥ 18 years of age with metastatic cancer in confirmed progression (up to 5 extracranial lesions) to a line of ICI (monotherapy or combined with other agents) while maintaining that same line due to clinical benefit [44], and referred for palliative SABR. The exclusion criteria included: (1) Eastern Cooperative Oncology Group (ECOG) performance status of 3 to 4; (2) brain metastases; (3) pseudo-progression or hyperprogression; (4) severe autoimmune diseases; or (5) previous RT that might interfere with the present treatment. Cohort B was a comparative group of metastatic patients receiving only SABR for oligorrecurrent or de novo OMD (up to five extracranial lesions). The study protocol was approved by the local ethics boards and followed the ethical standards of the Declaration of Helsinki [45]. All patients provided written informed consent before enrollment. For the cell-free DNA and cytometry analysis, we selected those patients in both cohorts with squamous and non-squamous NSCLC. We prioritized these patients due to NSCLC being the most frequent cancer type in both groups and, therefore, the most homogeneous subgroup. A third cohort of patients with only ICI is currently being recruited at our hospitals, and we expect to assess clinical and molecular differences in future analyses.

### 4.2. Clinical Protocol

The I-SABR protocol for cohort A closely follows that of our previous study on oligoprogression [10]. To summarize, we select 1 to 5 progressing and measurable lesions to treat and, if applicable, up to 2 nontarget lesions outside of the planned radiation field. Targets are chosen in the following order: (1) all symptomatic lesions (up to 5); (2) visceral/nodal lesions; and (3) bone lesions [46]. SABR is administered with palliative and immunostimulatory intent in 35 Gy in 5 nonconsecutive fractions (fx) (24 Gy in 3 fx in case of dose limitations). Systemic therapy is continued according to the standard protocol and administered concurrently with SABR and until further progression, unacceptable toxicity, or medical decision. Patients with bulky tumors > 65 cc are allowed to receive partial SABR in case of limiting dose constraints. Rechallenge with further SABR courses after new oligoprogression is allowed if the patient maintains clinical benefit, for a maximum of 3 courses. If further treatment is successful, we consider it an extension of clinical benefit and not a progression. Response is assessed by Immune Response Evaluation Criteria in Solid Tumors (iRECIST) [47] with computed tomography scanning (CT scan) 2 months after SABR and subsequently every 3 months. The treatment response before and after SABR was evaluated by 2 independent radiologists who were masked for the sites selected for measuring AR. For cohort B, SABR was administered in ablative doses to all visible lesions according to hospital protocol and depending on tumor site and dose constraints. The response was assessed using RECIST criteria with CT scanning 2 months after SABR and subsequently every 3 months. Given that there are currently few data on oligoprogression, the sample size was determined by assuming a 25% ORR with ICI alone in the metastatic population and an expected 42% ORR with I-SABR in our cohort, according to previously published results [5,10]. The null hypothesis for this study is that there is no significant difference at a molecular level between responders and non-responders in terms of ORR. The alternative hypothesis states that a significant molecular difference exists between the two groups. Considering a 20% drop out rate, a sample size of 72 patients for cohort A and another 72 patients in cohort B (for paired comparisons) was estimated to be enough to address the study’s potency to detect meaningful changes in the examined biomarkers with a 90% confidence interval and a power of 80%.

### 4.3. Translational Protocol

Blood samples were collected from patients in both cohorts at different time points: at patient recruitment (T1), following the first (T2) and last (T3) SABR fraction, two months after SABR (T4) and at further progression (TP).

For cfDNA analysis, samples were gathered in CellSave tubes (Menarini Silicon Biosystem Inc., Castel Maggiore, Italy) and then centrifuged at 1600 rpm for 10 min. The remaining plasma was centrifuged at 4750 rpm for 10 min. Plasma samples were stored at −80 °C in 3 mL cryovials. cfDNA was isolated from plasma with the QIAamp Circulating Nucleic Acid kit (Qiagen, Germantown, MD, USA, 55114) according to the manufacturer’s protocol. cfDNA was measured using 1X Qubit High Sensitivity (Thermo Fisher Scientific, Waltham, MA, USA). The fragment size, quality, and quantity of random samples were evaluated using a Bioanalyzer 2100 instrument (Agilent Technologies, Santa Clara, CA, USA).

Flow cytometry was performed in 10 mL of blood for each time point to assess the percentage of PBMCs with BD FACS Canto II cytometer (BD Biosciences, San Jose, CA, USA). Different PBMC subsets were characterized in 4 panels. Panel one was designed to study the ratio of T cells (CD3+) and specific cytotoxic CD8+ T cells (CD3+CD8+), antigen-presenting T cells expressing PD1 ligand (PDL1) (CD3+CD8+CD274+CD279−), and exhausted T lymphocytes (CD3+CD8+CD279+). In panel two, CD4+ T cells (CD4+) and regulatory T cells (CD4+CD25+CD127−) were evaluated. Panel three was designed to assess the ratio of natural killer cells (CD56+CD16−) and cytotoxic natural killers (CD56+CD16+/). Finally, in panel four, the ratio of activated B cells was measured in plasma (CD20+CD19+), long-lived plasma cells (CD138+CD27+) and memory B cells (CD19+CD20+CD27+). Sample data from all times were acquired with DIVA software (v9.0) and downloaded for detailed analyses by flowTOTAL [48].

EV analysis was conducted following the manual procedure of exoEasy Maxi Kit (QIAGEN, Venlo, The Netherlands). RNA isolation and small RNAseq sequencing were performed from plasma samples. microRNA-seq Data Standards and Processing Pipelines from ENCODE were used in combination with COMPRSA [49] for quality control, alignment, and annotation. Differential expression analysis of microRNAs (miRNAs) was performed employing the DESeq2 package [50]. Normalization and statistical analysis were carried out on the raw count data obtained from COMPSRA. A heat map was employed to visualize the expression patterns of miRNAs. Hierarchical clustering was utilized to group miRNAs (with *p* value < 0.05) and samples based on similarity in expression profiles. Relevant pathways were identified with ingenuity pathway analysis (IPA, QIAGEN) and those previously described in the literature.

### 4.4. Study Endpoints

The primary clinical endpoint is the last ORR at the time of analysis in terms of complete responses (CR) and partial responses (PR), which was evaluated in all sites of disease during follow-up. cfDNA concentration and small RNA differential expression is correlated with ORR at the different time points. Secondary endpoints include AR (cohort A), measured 2 months after SABR as a ≥30% reduction in the previously selected, nonirradiated lesions [51], PFS, overall survival (OS), local control (LC) (CR, PR, and stable disease), and toxicity (according to Common Terminology Criteria for Adverse Events version 5.0).

### 4.5. Statistical Analysis

Data were analyzed using SPSS version 26.0 (IBM) and RStudio 1.4 (https://cran.r-project.org, accessed on 1 July 2023) from 1 July to 30 November 2023. OS and PFS were estimated with the Kaplan–Meier method and compared between cohorts using the log-rank test. Data for patients who were alive were censored for OS at the time of the final follow-up. Data for those who were alive and had no tumor progression were censored for the evaluation of PFS and LC at the last assessment. To assess the differences between two numerical variables, the Wilcoxon rank-sum test (Mann–Whitney U test) was employed. For comparisons involving paired observations between two time points, the paired Wilcoxon signed-rank test was utilized. This test was applied to assess significant changes within paired data points, specifically examining increases or decreases in a variable over time. Principal component analysis (PCA) was employed to identify potential outliers in the dataset.

## 5. Conclusions

Our preliminary results reinforce the idea that I-SABR is an effective and safe approach in oligoprogression to ICI. There is a need for identifying biomarkers that can predict which patients have higher chances of responding to this approach. Our early analyses suggest that I-SABR can induce a decrease in the concentration of cfDNA that correlates with a good response. The increased presence of CD8+PD1+ cells and CD8+PDL1+ cells with SABR in non-responders and responders, respectively, is also an interesting finding that suggests the modulation of the immune response. Moreover, small RNAs including the RNU2 family, SNORD1B, hsa-miR-1-3p and hsa-miR-133a-3p are potential candidates in this setting. Further data from our study and other groups are needed to better define consistent biomarkers of response to I-SABR.

## Figures and Tables

**Table 1:** Patient and treatment characteristics (cohort A).

Characteristic	Value
**Gender**	
Male	14 (73.7%)
Female	5 (26.3%)
**Median age, years (range)**	67 (41–79)
**ECOG**	
0	10 (52.6%)
1	9 (47.4%)
**Primary tumor**	
Lung (non-squamous)	11 (57.8%)
Lung (squamous)	3 (15.8%)
Renal	3 (15.8%)
Bladder	1 (5.3%)
Cervix (squamous)	1 (5.3%)
**PD-L1 status (TPS)**	
<50%	3 (15.8%)
≥50%	9 (47.4%)
Unknown	7 (36.8%)
**Driver mutations**	
Yes	1 (5.3%)
No	18 (94.7%)
**Number of systemic therapy lines before SABR**	
None	12 (63.2%)
One	7 (36.8%)
**Prior radiotherapy**	
Yes	8 (42.1%)
No	11 (57.9%)
**Metastatic stage**	
Oligometastatic (1–5 lesions)	9 (47.4%)
Polymetastatic (>5 lesions)	10 (52.6%)
**Current systemic therapy**	
ICI only	15 (78.9%)
ICI plus chemotherapy	4 (21.1%)
**Primary resistance to ICI**	
Yes	4 (21.1%)
No	15 (78.9%)
**Irradiated tumor sites**	
Lung	10 (40%)
Nodes	6 (24%)
Bone	3 (12%)
Liver	2 (8%)
Other	4 (16%)
**Number of irradiated sites**	
One	13 (62%)
Two	6 (28%)
**SABR dose**	
35 Gy/5 fx	24 (98%)
24 Gy/3 fx	1 (2%)
**Partial SABR**	
Yes	3 (15.4%)
No	16 (84.2%)

Abbreviations: ECOG = Eastern Cooperative Oncology Group; SABR = stereotactic ablative radiotherapy; ICI = immune checkpoint inhibitor; fx = fractions; PD-L1 = programmed cell death ligand 1, TPS = tumor proportion score.

**Table 2:** Patient and treatment characteristics (cohort B).

Characteristic	Value
**Gender**	
Male	6 (65%)
Female	2 (25%)
**Median age, years (range)**	68 (38–73)
**ECOG**	
0	3 (37.5%)
1	5 (62.5%)
**Primary tumor**	
Lung (non-squamous)	4 (50%)
Renal	2 (25%)
Colorectal	1 (12.5%)
Liposarcoma	1 (12.5%)
**PD-L1 status (TPS)**	
<50%	2 (25%)
≥50%	1 (12.5%)
Unknown	5 (62.5%)
**Driver mutations**	
No	8 (100%)
**Number of systemic therapy lines before SABR**	
None	5 (62.5%)
One	2 (25%)
Two or more	1 (12.5%)
**Prior radiotherapy**	
Yes	4 (50%)
No	4 (50%)
**Metastatic stage**	
Oligometastatic (1–5 lesions)	7 (87.5%)
Polymetastatic (>5 lesions)	1 (12.5%)
**Irradiated tumor sites**	
Lung	6 (60%)
Nodes	2 (20%)
Bone	2 (20%)
**Number of irradiated sites**	
One	6 (75%)
Two	2 (25%)
**SABR dose**	
54 Gy/3 fx	2 (20%)
50 Gy/5 fx	4 (40%)
35 Gy/5 fx	1 (10%)
30 Gy/3 fx	1 (10%)
27 Gy/3 fx	1 (10%)
30 Gy/5 fx	1 (10%)
**Partial SABR**	
No	8 (100%)

Abbreviations: ECOG = Eastern Cooperative Oncology Group; SABR = stereotactic ablative radiotherapy; fx = fractions; PD-L1 = programmed cell death ligand 1, TPS = tumor proportion score.

**Table 3:** Treatment response.

Endpoint	N (%)	
	*Cohort A*	*Cohort B*
**Last Objective Response Rate (ORR)**	11/19 patients (57.9%)	6/8 patients (75%)
*Complete response*	4 (21.1%)	3 (37.5%)
*Partial response*	7 (36.8%)	3 (37.5%)
*Stable disease*	1 (5.3%)	0
*Progression disease*	7 (36.8%)	2 (25%)
**Abscopal response (AR) at 8 weeks**		
*Present*	5/14 patients (35.7%)	NA
*Absent*	9/14 patients (64.3%)	NA
**Local control (LC)**	22/25 lesions (88%)	10/10 lesions (100%)
*Complete response*	12 (48%)	7 (70%)
*Partial response*	8 (32%)	3 (30%)
*Stable disease*	2 (8%)	0
*Progression disease*	3 (12%)	0

## Data Availability

Research data are stored in an institutional repository and will be shared upon reasonable request to the corresponding author.
